# Conjugation of Short-Chain Fatty Acids to Bicyclic-Amines for Analysis by Liquid Chromatography Tandem Mass Spectroscopy

**DOI:** 10.3390/molecules30020341

**Published:** 2025-01-16

**Authors:** Daniel N. Darlington

**Affiliations:** 1 Blood and Shock Research, US Army Institute of Surgical Research, JBSA Fort Sam Houston, San Antonio, TX 78234, USA; daniel.n.darlington.civ@health.mil; 2 Department of Surgery, Trauma and Emergency Surgery, University of Texas Health, San Antonio, TX 78229, USA

**Keywords:** short-chain fatty acids, chromatography, mass spectroscopy, conjugation, plasma, liver, platelets, red blood cells

## Abstract

Conjugation of short-chain fatty acids (SDFAs) to amines containing ring structures allows for better measurement by liquid chromatography tandem mass spectroscopy (LC-MS/MS). However, collision-induced dissociation (CID) results in breaking the conjugate back to the original SCFA and amine. We therefore set out to find an amine that would remain on the SCFA after CID and create a unique daughter for selectivity of measurement. Of twenty-seven amines with ring structures, we found four that contain bicycle-type structures (two rings connected by a carbon) with nitrogen in the second ring. CID removes the second ring at the nitrogen, leaving the first ring on the daughter. Of the four amines, 4-(pyrrolidine-1-ylmethyl) benzylamine (4PyBA) showed the strongest conjugation. Conjugation of 4PyBA to SCFA (C3–C6), their isomers and their phenylated versions (and isomers) resulted in good chromatographic peaks and separation. CID resulted in unique daughters that allowed for selectivity of measurement. Using this method, standard curves were generated that show good linearity (r2 > 0.99) in the nM and μM range with lower limits of detection between 40 and 229 nM for a 10 μL sample. Finally, we used this method to measure SCFA in plasma, liver, platelets, and red blood cells, demonstrating its use in biological systems. Because SCFAs are an index of microbiome diversity in the gastrointestinal track, this method will allow us to study changes in SCFAs and the microbiome in pathologic conditions including trauma, hemorrhage, and sepsis.

## 1. Introduction

Short-chain fatty acids (SCFAs) are naturally occurring carboxylic acids with aliphatic chains (C1–C6) and are the main fermentation product of dietary fiber by intestinal microbes. They are involved in many metabolic processes such as ATP production, modulation of inflammation and immune responses [1,2,3,4,5], as well as in the pathogenesis of diseases such as Parkinson’s disease [6], Alzheimer’s disease [7,8], inflammatory bowel disease [9], and liver disease [10]. The human gut microbiome produces various SCFAs through the degradation of carbohydrates and amino acids [1,11,12,13]. SCFA supplementation has been suggested for improving outcome in liver disease [10] and endometriosis [14] and spinal cord trauma [15]. Because of the recent interest in research on the impact of the microbiome on health and disease, the measurement of SCFAs in blood and tissue has become a key element in the study of the gut microbiome. However, the measurement of SCFAs is difficult and has been almost completely performed by mass spectroscopy.

Tandem mass spectroscopy (MS/MS) is a powerful tool that allows for the measurement of analytes with great specificity. The most common tandem mass spec configurations use three linear accelerators. The first acts as a filter when used in selective reaction monitoring (SRM) mode, to select specific parent analytes by their molecular weight. These analytes are “filtered” into the second linear accelerator, which is filled with collision partials (i.e., argon gas). The analytes collide with the argon and create daughters or transitions (collision-induced dissociation, CID). These daughters tend to be unique to the parent and add a degree of selectivity or specificity to the measurement. The daughters then pass to the third linear accelerator where they are measured in the SRM mode. The measurement of the daughter represents the measurement of the parent. The addition of liquid chromatography (LC) at the front end of the MS/MS (LC-MS/MS) allows for even greater selectivity as analytes can be separated in time prior to entering the MS. This is essential for analytes that contain many isomers, like SCFA, or analytes of similar chemical configuration.

LC-MS/MS is increasingly becoming a standard in the measurement of small-molecular-weight analytes (<2000 Da). However, for MS/MS to work, the analyte must be charged (+ or −) prior to entry into the MS. Very small-molecular-weight and linear carbon structures are difficult to charge (or protonate) using the various ionizing methods (for example, electrospray, matrix-assisted laser desorption/ionization—MALDI). SCFAs do not charge readily and are therefore difficult to measure with MS. The problem has been solved with the conjugation of the SCFAs to amines [16] with ring structures (ex, phenyl, pyridine, and pyrimidine). Ring structures ionize or protonate easily, and the final conjugate can be detected.

However, CID in the second linear accelerator breaks the conjugate back to the original parent and the amine. In the case of SCFAs, conjugation to an amine results in strong conjugation to a new parent molecule (SCFA-amine). But after CID, the conjugate dissociates back to the original SCFA and amine. But because the amine is in a 1:1 molar ratio, the amine is measured as a proxy for the SCFA as it has the strongest signal. This method has been used by many researchers [16,17,18,19,20,21,22,23,24], including us (unpublished data).

Although successful, selection of the proxy for measurement of the original analyte has a theoretical issue that needs to be addressed. The method combines carboxylic acid (SCFA) with an amine, and the reaction is not specific for SCFA, as any carboxylic acid in the mixture will conjugate to the amine. Biological samples (plasma or tissue) contain many classes of molecules with carboxylic acids, and they will conjugate with the amine along with the SCFA. These “other” conjugates have the potential to interfere with measurement of the SCFA conjugate because, in the end, the MS is detecting and measuring the amine, not the SCFA. The results could lead to false positive or falsely elevated levels of SCFA. 

In an attempt to circumvent this problem, we have searched for ring structured amines that would conjugate to the SCFA and leave a portion of the conjugate on the daughter molecule during collision-induced dissociation. This would result in a daughter that is truly unique to the parent and could be used for selective measurement. To this date, we have screened 27 different amines (with ring structures) for both strong conjugation to SCFA and the ability to create a unique daughter. Of these twenty-seven, we found four. Using the one with the strongest conjugation, we developed a method to measure SCFA (C2–C6 and all the isomers) in plasma and various tissues. Our goal was to create daughters that still have a portion of the amine connected to the SCFA and allow for measurement specificity.

## 2. Results

### 2.1. Selection of Amine Types for Conjugation

We conjugate four SCFA (propionic, butyric, pentanoic, and hexanoic acid) to twenty-seven different amines with different configurations (Table 1) by the methods described below. These amines were chosen for three reasons: (1) they had simple or complex ring structures that would enhance chromatographic separation, (2) they could be easily protonated by the electrospray ionization thereby generating a greater signal from the conjugated SCFA compared to the unconjugated SCFA, and (3) they had chemical side groups that could (potentially) be removed after collision-induced dissociation with argon, and result in a portion of the amine conjugated to the daughter.

This last reason is important as we have found that the conjugation of SCFA and amines performed in our lab and others [17,18,19,20,21,22,23,24] fell apart after CID into the original analytes (SCFA and amine).

**Figure 1:**
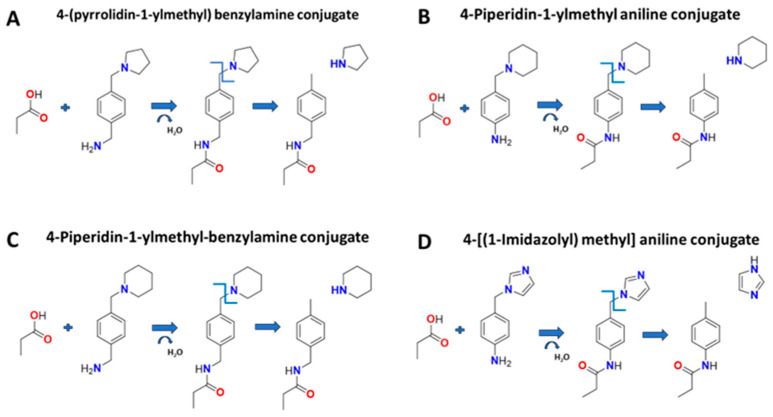
Conjugation of propionic acid to four different bicycle amines (**A**–**D**). The conjugation is a dehydration reaction (loss of H_2_O first arrow in each caption). The resulting propionate-amine conjugate (parent molecule) enters the MS/MS for collision-induced dissociation creating a daughter molecule with a benzyl ring (2nd arrow each caption). Each daughter contains the original propionate and part of the amine, resulting in a unique daughter for the selectivity of measurement.

All amines in Table 1 conjugated to the four SCFA to various degrees of efficiency. However, four amines had both a high conjugation efficiency (Table 1, bold with asterisk), and a portion of the conjugate remains on the daughter after collision-induced dissociation. These amines, their conjugates to SCFA, and their resultant daughter structures are shown in Figure 1. All four amines showed excellent separation on reverse phase chromatography. These amines had a bicycle configuration that included two ring structures connected by carbon. The second ring was connected by nitrogen. We found that the parent conjugate dissociated at the nitrogen on the second ring. The lack of nitrogen on the second ring, or the substitution of the connecting carbon with sulfur or oxygen (4-phenylthio aniline, 3-phenoxy-benzylamine, 3-pyridine-2-yloxy benzylamine, 4-p-tolyoxy-benzylamine) did not produce strong conjugation or did not break at the second ring.

To determine which of the four bicycle-amines had the strongest signal, we conjugated propionic, butyric, pentanoic, and hexanoic acid to the four bicycle amines under the same experimental conditions and ran them on LC-MS/MS. Each amine conjugate had methods developed that monitored the unique precursor and products (daughters) in single reaction monitoring mode, and the parameters are in Table 2. The results showed that 4-(pyrrolidine-1-ylmethyl) benzylamine (4PyBA, case # 91271-79-3, PubChem) had the highest signal (area under the curve) for each conjugate. Therefore, in this study, we chose 4PyBA conjugate amine to proceed. All other amines in Table 1 contained side groups from the primary ring structure that were hypothesized to be removed after CID, leaving the ring connected to the daughter. However, this did not happen with any of the other amines in Table 1. Most of them conjugated to the SCFA but broke down after CID into the original SCFA and amine. These data suggest that nitrogen is the weak point for dissociation in the argon gas, and the nitrogen in the second ring tends to be the weak link resulting in a daughter bound to the amine (Figure 1).

### 2.2. Conjugation of 4PyBA to SCFA

Using 4PyBA, we conjugated all SCFAs (C3–C6 with all isomers, and phenyl SCFA with isomers). Some of the proposed conjugates are shown in Supplement Figure S1. Table 3 shows the molecular weights for the original SCFA, the parent conjugate (SCFA-4PyBA), and the daughter conjugates. Note that the daughter conjugate is smaller than the parent conjugate but bigger than the original SCFA, demonstrating the loss of the second ring, as illustrated in Figure 1.

**Figure 2:**
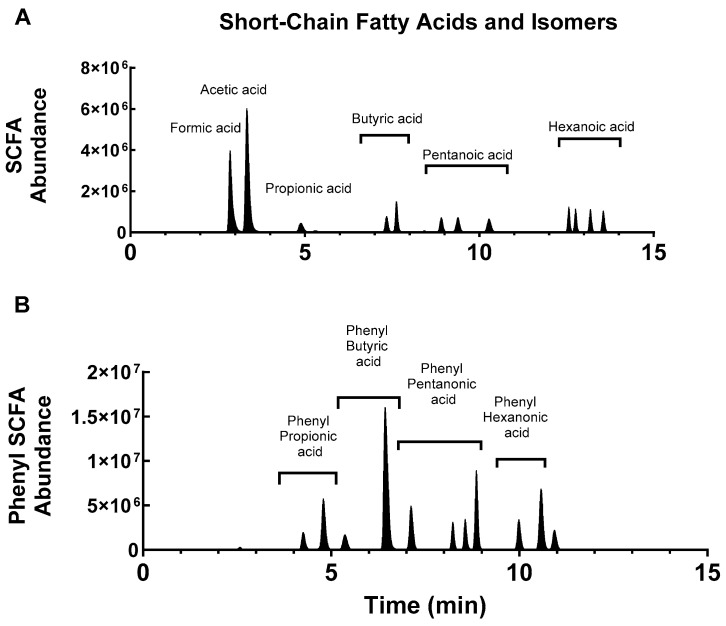
Total ion chromatograms of SCFA-4PyBA conjugates (**A**) and phenyl-SCFA-4PyBA conjugates (**B**). All isomers were separated except for 2- and 4-phenyl butyric acid (largest peak in the phenyl-butyric acid group). These peaks overlapped and could not be separated.

**Figure 3:**
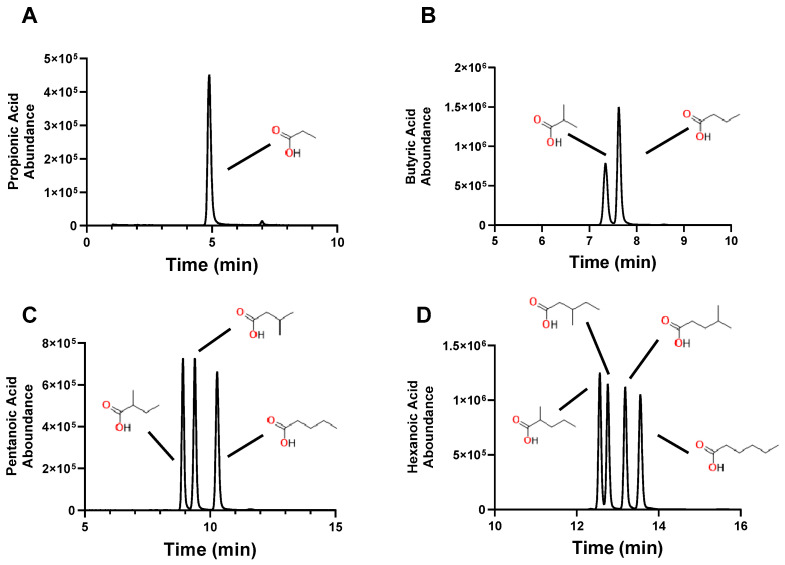
Individual chromatograms (multiple ion monitoring) from C3 (**A**), C4 (**B**), C5 (**C**), and C6 (**D**) SCFA-4PyBA conjugates. Notice the excellent separation of isomers. Chemical structures of original SCFAs are included for clarity. The area under the curve (peak) is proportional to the amount of SCFA.

**Figure 4:**
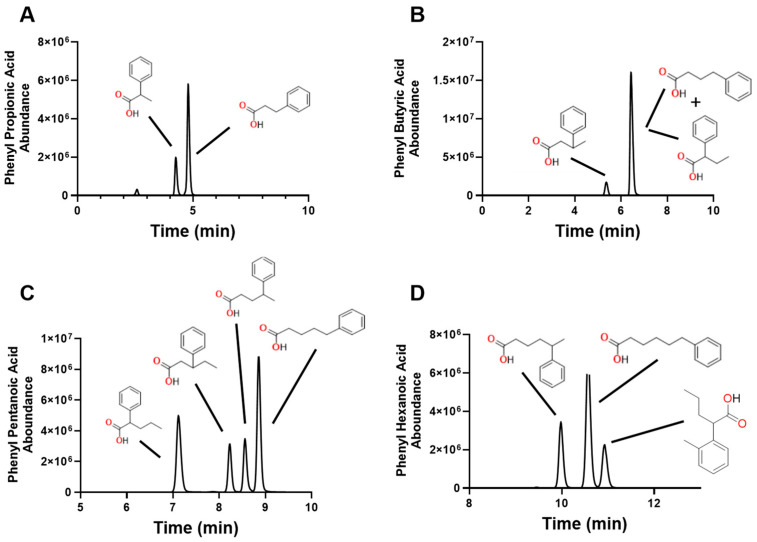
Individual chromatograms (multiple ion monitoring) from phenylated C3 (**A**), C4 (**B**), C5 (**C**), and C6 (**D**) SCFA. Notice the excellent separation of isomers, except for 2- and 4-phenyl butyric acid. Chemical structures of original phenyl-SCFA are included for clarity. The area under the curve (peak) is proportional to the amount of phenyl-SCFA.

Chromatographic separation of the SCFA and their isomers are shown in Figure 2. The separation was confirmed by injecting individual SCFA-4PyBA conjugates into the LC-MS/MS system. We were able to easily distinguish all isomers, as shown in Figure 3, and for the phenyl-SCFA, as shown in Figure 4. Only two isomer peaks overlapped in chromatography (Figure 4, 2- and 4-phenyl butyric acid). Standard curves were generated by varying the dose for all SCFAs and their isomers (Table 4). The standard curves show that the SCFAs can be detected in the nM and μM range for a 10 μL sample. The standards showed good linearity (r2 > 0.99). Accuracy is shown by the lower limit of detection (LOD) that varied between 40 and 229 nM, and precision by percent coefficient of variation for each analyte at 1 μM and 100 nM (Table 4).

### 2.3. Measurement of SCFA in Biological Samples

**Figure 5:**
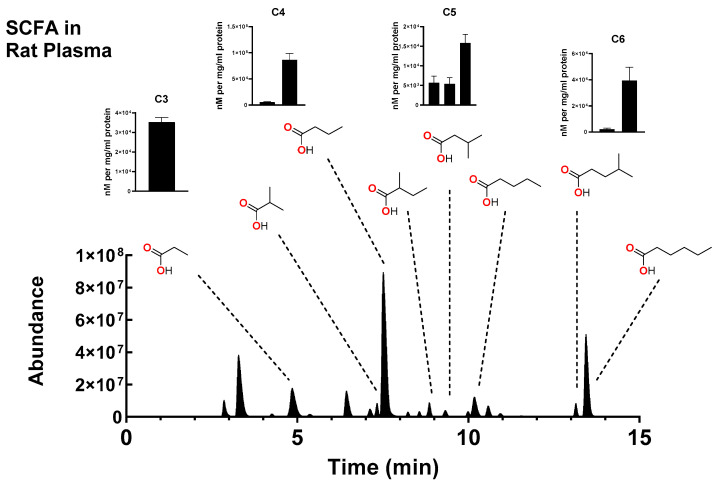
Representative total ion chromatogram of SCFA-4PyBA conjugates derived from rat plasma. The SCFAs represented by the peaks are included (chemical structures). The area under the peaks from 7 rats were analyzed and compared to a standard curve. nM amounts were calculated for the 7 rats. BCA protein assays were performed on each sample to correct for mg of protein.

Figure 5 shows the chromatogram of various SCFA identified in extracted rat plasma. The area under each peak was analyzed and compared to standard curves for calculation of the concentration in nM (Table 4). Identification of each SCFA was performed by (1) matching peaks in the plasma sample to the standards, (2) measuring the area under the curves, and (3) fitting that area to standard curves for the final measurement of each SCFA. Using this method, we were also able to detect SCFA and phenyl SCFA in pig liver, rat platelets, and rat red blood cells (Supplementary Figures S2–S7), demonstrating the use of this method in different species and tissues.

## 3. Discussion

The gut microbiome regulates gut barrier function, immunity, and the intake of metabolic nutrients [2]. SCFAs found in the gut are considered a marker for a healthy gut ecosystem [2], and the measurement of SCFAs could be used as an index of microbiome health, and therefore overall health. Changes in the gut microbiome and SCFAs are associated with inflammation and immune responses [1,2,3,4,5], Parkinson’s disease [6], Alzheimer’s [7,8], inflammatory bowel disease [9], and liver disease [10]. We have shown that trauma and hemorrhage change the beta diversity of gut bacteria in rats [25], suggesting that SCFA composition may change as well. This current method will allow us to measure the changes in SCFA after trauma and hemorrhage, and other pathophysiological conditions.

We have shown that SCFAs can be successfully conjugated to four bicycle amines. The strongest conjugation was observed with 4-(pyrrolidin-1-ylmethyl) benzylamine (4PyBA), and this conjugation has led to a daughter that contained a fragment of the conjugate. This allows for better selectivity of the analyte and less chance for interference by other carboxylic acids found in biological fluid or tissue. Because the conjugate contains a ring structure, the signal for detection by MS is strong. This method shows excellent chromatographic peaks, and good separation of isomers. This is important as SCFAs may have many isomers that can interfere with measurements. Standard curves can be generated and show good linearity in the nM and μM range. Finally, this method can be used to measure SCFA quantitatively in plasma and tissues.

LC-MS/MS is a powerful tool and standard in the industry to measure small-molecular-weight analytes. However, not all analytes can be detected. SCFAs are an example, as their linear structure does not allow them to be easily “charged” for MS detection. Conjugation to amines [16] is easily ionized, and the final conjugate can be detected. Derivatives used for SCFA conjugation include 4-aminomethylquinoline [17], 2-picolylamine [18], 3-nitrophenylhydrazine [19,21], 4-acetamido-7-mercapto-2,1,3-benzoxadiazole [26], and O-benzylhydroxylamine [22,24]. However, collision-induced dissociation breaks the conjugate back to the original parent and the amine. The amine is then measured as a proxy for the SCFA.

We have found that conjugation to ring structures allows the final conjugate to be charged, leading to better MS detection, chromatographic separation, and the final transition still contains a part of the conjugate allowing for better specificity of measurement. We have used a class of “bicycle-amines” to measure a class of carboxylic acids that normally cannot be detected on MS in the unconjugated form. It is very possible that this method could be expanded to detect other carboxylic acids, besides SCFA, that are difficult to detect by MS, or any other method. For example, we have had some success modifying the method to measure lactate and pyruvate, although the method does not work well with citrate or isocitrate (unpublished data). These are carboxylic acids that are important in metabolism and ATP generation but are very difficult to measure. However, we believe that this example illustrates the potential use of this method, and that the method could be expanded to detect other analytes that are difficult or impossible to measure.

## 4. Materials and Methods

### 4.1. Materials

All chemicals were purchased at the highest quality. 2′,2′-dipyridyl disulfide, triphenylphosphene, formic acid, acetic acid, propionic acid, 2-phenyl propionic acid, 3-phenyl propionic acid, butyric acid, 2-methyl propionic acid, 2-phenyl butyric acid, 3-phenyl butyric acid, 4-phenyl butyric acid, pentanoic acid, 2-methyl butyric acid, 3-methyl butyric acid, 4-methyl butyric acid, 2-phenyl pentanoic acid, 4-phenyl pentanoic acid, 5-phenyl pentanoic acid, hexanoic acid, 2-methyl pentanoic acid, 3-methyl pentanoic acid, 4-methyl pentanoic acid, 5-phenyl hexanoic acid, 6-phenyl hexanoic acid, acetone, dichloromethane, ethyl acetate, 1-butanol, ammonium formate, ammonium acetate, ammonium carbonate, 3-methoxytyramine, 3-hydroxytyramine, tyramine, normetanephrine, 5-hydroxy tryptamine, 5-methoxy tryptamine, 5-benyloxy tryptamine, tryptamine, 3,4-dihyroxybenzylamine, 3-nitrophenyl hydrazine, 2,4-dinitrophenyl hydrazine, dansyl hydrazine, 4-aminomethylquinoline, 4-methoxyphenethylamine, 4-(1H-imidazol-1-yl) aniline, 2-picolylamine, 3-picolylamine, and O-benzylhydroxylamine HCl were purchased from MilliporeSigma (Burlington, MA, USA). 3-phenyl pentanoic acid, 6-phenyl hexanoic acid, and 4-piperidin-1-ylmethyl aniline were purchase from Smolecule (San Antonio, TX, USA). 4-(pyrrolidin-1-ylmethyl) benzylamine was purchased from Santa Cruz Biotechnologies (Dallas, TX, USA). 3-(pyridin-2-yloxy) benzylamine was purchased from AstaTech Inc. (Bristol, PA, USA). 3-phenoxy-benzylamine was purchased from MatrixScienfic (Columbia, SC, USA). 4-p-tolyloxy-benzylamine was purchased from Achemblock (Hayward, CA, USA). 4-[(1-imidazolyl) methyl] aniline, 3-(piperidin-1-yl) benzylamine, 4-(1H-pyrrol-1-yl) benzylamine, 4-[(1-imidazolyl) methyl] aniline, and 4-(phenylthio) aniline was purchased from Boc Sciences (Shirley, NY, USA). Acetonitrile (MeCN) was purchased from ThermoFisher (Waltham, MA, USA). Not all Phenyl SCFAs are represented as they could not be purchased.

### 4.2. Conjugation Reaction

All reactions were performed in a siliconized glass 12 × 75 tube with 10 μL of SCFA (100 μM in MeCN), 50 μL of triphenylphosphene (10 mM in MeCN), 50 μL 2,2′-Dipyridyl disulfide (10 mM in MeCN), and 50 μL of amine (10 mM in MeCN). The mixture was incubated for 10 min at 60 °C, diluted with 2.7 mL ddH2O, placed over HLB (Waters) solid-phase extraction columns, washed with 5% MeOH, and eluded in 100% MeOH. The eluant was dried by spin dryer. The dried eluant was brought up in 200 μL 5% MeCN, 0.1% formic acid and analyzed by LC-MS/MS (Ultimate 3000-Quantiva, ThermoFisher).

### 4.3. LC-MS/MS

SCFA conjugates were separated by reverse phase chromatography on a Kinetex Polar C18 150 × 2.1 mm column from Phenomenex (Torrance, CA, USA) at 250 μL/mL. A stepwise gradient was used, starting at 5% MeCN, 0.1 formic acid, 12% at 4 min, 20% at 9 min, 100% at 13 min and back to 5% at 15 min. Mass spectroscopy (Quantiva, ThermoFisher) was performed using a triple quadruple configuration (Q1, Q2 and Q3) in selective reaction monitoring mode in both Q1 (for section of the parent SCFA conjugates) and Q3 (for selection of daughters). Q2 contained argon gas (1.5 Torr) for collision-induced dissociation of parent to daughter. All samples are run at 10 μL in duplicate or triplicate. Heated-electrospray ionization (vaporizing temperature 100 °C) was used to ionize the analytes prior to entering the MS (spray voltage 5000 V, ion transfer tube 275 °C, sheath gas 15, sweep gas 2, and auxiliary gas 5 torr).

### 4.4. Screening of SCFA Conjugation to 27 Different Amines

Propionic, butyric, pentanoic, and hexanoic acids were conjugated to 27 different amines (Table 1) by the method described above. Each conjugate was scrutinized for the proof of conjugation (correct molecular weight for the predicted SCFA conjugate) and if the daughter was still bound to part of the amine.

### 4.5. Extraction of SCFAs from Plasma and Tissue

In order to measure SCFAs from plasma and tissues, we spiked human plasma and pig liver slurry with various SCFAs and used 20 different extraction methods to determine optimal extraction. A solution of SCFA was created (100 μM in MeCN) that included propionic acid, butyric acid, isobutyric acid, pentanoic acid and its two isomers (2 and 3 methyl butyric acid), and hexanoic acid and its 3 isomers (2,3, and 4 methyl pentanoic acid). A 2nd solution of phenylated SCFA was also created (100 μM in MeCH) and included 2- and 3-phenyl propionic acid, 2-,3- and 4-phenyl butyric acid, 2-,3-,4- and 5-phenyl pentanoic acid, and 5- and 6-phenyl hexanoic acid.

Liver tissue was obtained from recently deceased swine in a tissue sharing agreement in the Institute of Surgical Research. On ice, 1 g of tissue was mixed with 5 mL of phosphate-buffered saline, homogenized, sonicated and homogenized again until a fine slurry was formed. Human whole blood was collected from the Institute of Surgical Research Blood Bank (L-18-008), centrifuged at 3000× *g* for 10 min and the plasma removed.

In total, 100 μL of human plasma or 100 μL of liver slurry was placed in twenty 1.5 mL tubes with 10 μL of SCFA or phenyl-SCFA solutions. The SCFA/tissue/plasma mix was vortexed. The SCFA and phenyl-SCFA were extracted from the liver or plasma by 20 different solutions including 1 mL each of EtOH, MeOH or MeCN with or without 100 mM of formic acid, ammonium formate, ammonium acetate or ammonium carbonate. Also included were EtOH:MeOH:MeCN 1:1:1, ethyl acetate, acetone, dimethyl chloride or 4-butanol. Each tube was vortexed, centrifuged (5000× *g* for 5 min) to remove precipitated protein, and the supernatant (or organic phase) was dried by centripetal dryer (ThermoFisher). These samples were bought up in 200 μL of 5%MeCN, 0.1% formic acid, and run on LC-MS/MS (10 μL) to determine which extraction method gave the highest levels of SCFA or phenyl-SCFA.

Rat whole blood was also obtained from the Institute of Surgical Research tissue sharing agreement. The blood was centrifuged at 200× *g* for 10 min, with no breaks, to separate platelet-rich plasma from red blood cells. The platelet-rich plasma was further centrifuged (5000× *g* for 1 min) and a platelet pellet was separated from the plasma and washed 3× with PBS.

### 4.6. Extraction and Conjugation

In total, 100 μL of rat plasma, 100 μL of rat red blood cells, the rat platelet pellet or 100 μL pig liver slurry were extracted with 1 mL of MeCN, vortexed, and the supernatant dried. The dried extraction derived from red blood cells, platelets, plasma or liver was then used in the conjugation procedures described above. 8-bromo-adenosine was used as an internal control for all samples to monitor the extraction and measurement efficiency throughout the process. The same amount of internal control was added to all samples including the standards.

### 4.7. Data Analysis

For standard curves, linear regression analysis was performed for calculation of slope, interception, and correlation coefficient. The lower limit of detection (LOD) was calculated as 3.3 times the standard deviation of the response divided by the slope. Mean and standard error of the mean (SEM) were calculated for the measurement of SCFA extracted from the plasma of a group of 7 rats. The % coefficient of variation was calculated as the SD/mean × 100%.

## 5. Conclusions

We have shown that SCFAs can be successfully conjugated to four bicycle amines. The strongest conjugation was observed with 4-(pyrrolidin-1-ylmethyl) benzylamine (4PyBA), and this conjugation has led to a daughter that contained a fragment of the conjugate. This allows for better selectivity of the analyte and less chance for interference by other carboxylic acids found in biological fluid or tissue. Because the conjugate contains a ring structure, the signal for detection by MS is strong and the chromatography shows good separation of isomers. This current method will allow us to measure the changes in SCFAs in tissues and plasma after trauma and hemorrhage, and other pathophysiological conditions.

## 6. Patents

This project supports the provisional patent Docket Number 15969-048US0.

## Figures and Tables

**Table 1:** Amines used for conjugation to SCFA.

*Amino pyridines*	*Phenyl amines*
2-picolylamine	3,4-dihyroxybenzylamine
3-picolylamine	O-benzylhydroxylamine
*Tyramines and tryptamines*	*Hydrazine*
3-methoxytyramine	3-nitrophenyl hydrazine
tyramine	2,4-dinitrophenyl hydrazine
normetanephrine	dansyl hydrazine
5-hydroxy tryptamine (serotonin)	
5-methoxy tryptamine	*Phenyl ethyl amines*
5-benyloxy tryptamine	4-methoxyphenethylamine
tryptamine	
	*Quinoline*
*Benzyl Amines*	4-aminomethylquinoline
4-(pyrrolidin-1-ylmethyl) benzylamine *	
4-piperidin-1-ylmethyl-benzylamine *	*Anilines*
4-(1H-Pyrrol-1-yl) benzylamine	4-[(1-imidazolyl) methyl] aniline *
3-(piperidin-1-yl) benzylamine	4-piperidin-1-ylmethyl aniline *
3-phenoxy-benzylamine	4-(1H-imidazol-1-yl) aniline
3-(pyridin-2-yloxy) benzylamine	4-(phenylthio) aniline
4-p-tolyloxy-benzylamine	

All amines were conjugated to short-chain fatty acids (propionic, butyric, pentanoic, hexanoic acid) under the same conditions. All conjugated products were analyzed in Q1 of MS/MS, and their transitions (daughters) analyzed in Q3. Conjugates that had both strong Q1 signals (parent conjugate) and strong Q3 signals (daughter conjugate) are presented with asterisk (*).

**Table 2:** SCFA-bicycle amine conjugates.

(AUC)	Propionic	Butyric	Pentanoic	Hexanoic
4PyBA	8.44 ± 0.20	7.30 ± 0.26	4.48 ± 0.12	3.59 ± 0.03
4PpBA	6.58 ± 0.06	6.50 ± 0.03	0.47 ± 0.09	2.93 ± 0.15
4PMA	1.84 ±0.04	2.14 ± 0.03	1.17 ± 0.01	1.45 ± 0.01
4IMA	1.02 ± 0.09	1.32 ± 0.11	0.62 ± 0.04	0.51 ± 0.01
	**Precursor (*m*/*z*)**			
4PyBA	247.1	261.2	275.2	289.2
4PpBA	261.2	275.3	289.3	303.2
4PMA	247.2	261.2	275.3	289.3
4IMA	230.2	244.2	258.2	272.2
	**Product (*m*/*z*)**			
4PyBA	176.0	190.1	204.3	218.1
4PpBA	176.1	190.4	204.0	218.1
4PMA	162.1	176.0	190.0	204.1
4IMA	162.1	176.0	190.1	204.1

The area under the curve (AUC) values for conjugates of SCFA (propionic, butyric, pentanoic, and hexanoic acid) to the four bicycle amines. Values represent mean and SEM of 4 replicates. Also included are the molecule weights (*m*/*z*) of the precursors (parent conjugate) and products (daughters) used in the measurement by LC-MS/MS.

**Table 3:** Molecular weights (MW) of the original analytes (and isomers), the conjugate parent (4PyBA-SCFA), and conjugate daughter. Notice that the conjugate daughter is larger than the original analyte, but smaller than the conjugate parent demonstrating that part of 4PyBA is still conjugated to the SCFA after collision-induced dissociation.

	MW Analyte	MW Conjugate-Parent	MDW Conjugate-Daughter
Propionic acid	74	247	176
Butyric acid and C4 isomers	88	261	190
Pentanoic acid and C5 isomers	102	275	204
Hexanoic acid and C6 isomers	116	289	218
Phenyl-propionic acid (2 isomers)	150	323	254
Phenyl butyric acid (3 isomers)	164	337	266
Phenyl pentanoic acid(4 isomers)	178	351	280
Phenyl hexanoic acid (2 isomers)	192	365	294

**Table 4:** Standard curves.

	Linear Range (nM)	r2	LOD (nM)	%CV 1 μM	%CV 100 nM
C3					
Propionic acid	1–3333	0.9982	227	1.25	4.26
2 Phenyl Propionic acid	1–3333	0.9994	69	2.09	6.87
3 Phenyl Propionic acid	1–3333	0.9995	117	1.95	3.44
C4					
Butyric acid	1–1000	0.9876	187	1.46	4.61
2 Methyl Propionic acid	1–3333	0.9993	135	0.34	3.97
2/4 Phenyl Butyric acid	1–1000	0.9994	40	2.18	6.62
3 Phenyl Butyric acid	1–1000	0.9993	43	2.55	9.18
C5					
Pentanoic acid	1–1000	0.9816	229	1.48	5.71
2 Methyl Butyric acid	1–1000	0.9970	92	1.40	6.12
3 Methyl Butyric acid	1–1000	0.9993	165	1.28	6.10
2 Phenyl Pentanoic acid	1–1000	0.9992	46	2.34	3.96
3 Phenyl Pentanoic acid	1–1000	0.9993	43	1.80	3.02
4 Phenyl Pentanoic acid	1–1000	0.9994	40	2.07	4.12
5 Phenyl Pentanoic acid	1–1000	0.9981	51	2.00	4.26
C6					
Hexanoic acid	1–1000	0.9966	114	1.49	4.96
2 Methyl Pentanoic acid	1–1000	0.9975	84	1.13	5.09
3 Methyl Pentanoic acid	1–1000	0.9980	76	0.92	5.86
4 Methyl Pentanoic aci	1–1000	0.9973	88	1.88	5.16
5 Phenyl Hexanoic acid	1–1000	0.9986	63	4.79	4.33
6 Phenyl Hexanoic acid	1–1000	0.9992	48	0.88	4.66

Short-chain fatty acids (SCFAs) are grouped by the number of aliphatic carbons in the base chain. Phenyl functional groups are connected to carbons numbered from the carboxylic acid of the base chain. 2- and 4-phenyl butyric acid could not be separated chromatographically so are reported together. Correlation coefficient (r2) and the lower limit of detection (LOD) are calculated from the entire range. LOD = 3.3 × (standard deviation of the response/slope). The injection volume was 10 μL. %CV = SD/mean of three replicates × 100% for each analyte at concentrations 1 μM and 100 nM.

## Data Availability

The authors will make data available after DOD review of request.

## Supplementary Materials

The following supporting information can be downloaded at: https://www.mdpi.com/article/10.3390/molecules30020341/s1, Figure S1: Schematics for conjugation of various SCFA to 4PyBA; Figure S2: Chromatographs showing separation of C3, C4, C5 and C6 SCFA-4PyBA in pig liver; Figure S3: Chromatographs showing separation of C3 and C4 phenyl-SCFA-4PyBA in pig liver; Figure S4: Chromatographs showing separation of C3, C4, C5 and C6 SCFA-4PyBA in rat platelets; Figure S5: Chromatographs showing separation of C3, C4 and C6 phenyl-SCFA-4PyBA in rat platelets; Figure S6: Chromatographs showing separation of C3, C4, C5 and C6 SCFA-4PyBA in rat red blood cells; Figure S7: Chromatographs showing separation of C3, C4 and C6 phenyl-SCFA-4PyBA in rat red blood cells.

## Abbreviations

The following abbreviations are used in this manuscript:

CID	collision-induced dissociation
EtOH	ethanol
ESI	electrospray ionization
MS	mass spectroscopy
MeCN	acetonitrile
MeOH	methanol
LC-MS/MS	liquid chromatography tandem mass spectroscopy
Q	quadrupole (configuration of a single linear accelerator in MS/MS)
SRM	selective reaction monitor (or single ion monitoring, multiple ion monitoring)
SCFAs	short-chain fatty acids
4PyBA	4-(pyrrolidin-1-ylmethyl) benzylamine
MALDI	matrix-assisted laser desorption/ionization
