# Supplementary material for: Conjugation of Short-Chain Fatty Acids to Bicyclic-Amines for Analysis by Liquid Chromatography Tandem Mass Spectroscopy
Source: Molecules. 2025 Jan 16;30(2):341. doi: 10.3390/molecules30020341 (PMC11768009; doi:10.3390/molecules30020341)
Supplement: Supplementary file 1 [file molecules-30-00341-s001.zip › molecules-3410562-supplementary.pdf]

Supplementary Figures:

Figure S1.

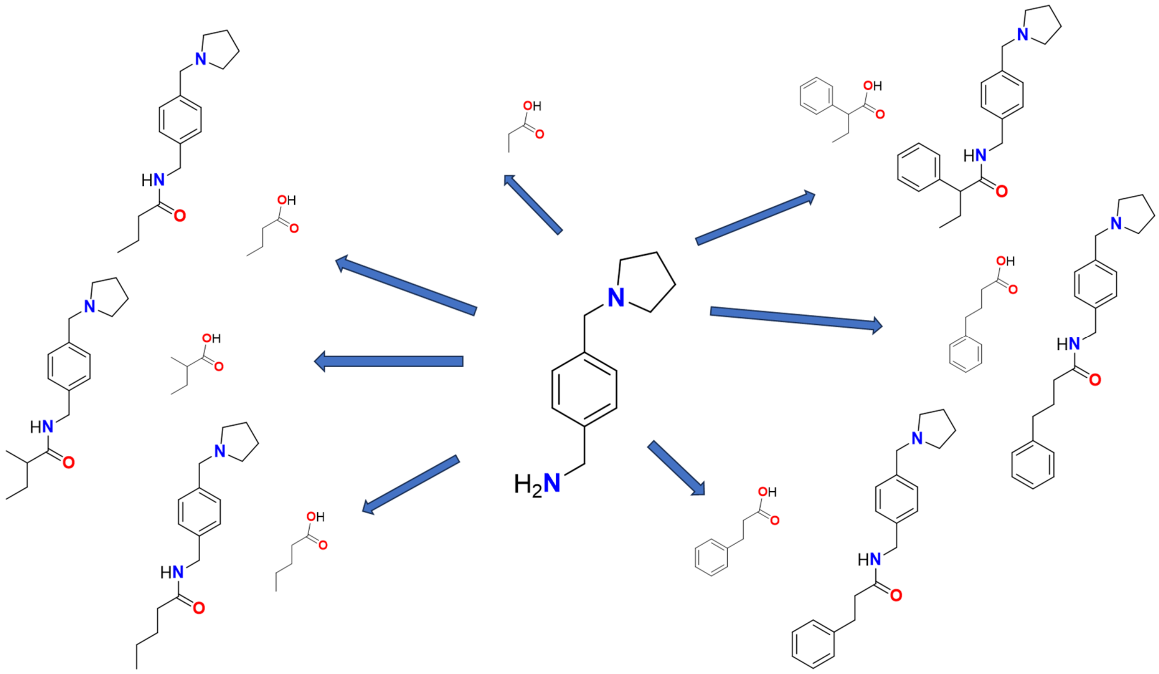

Supplementary Figure S1. Schematics for conjugation of various SCFA to 4PyBA.

Figure S2.

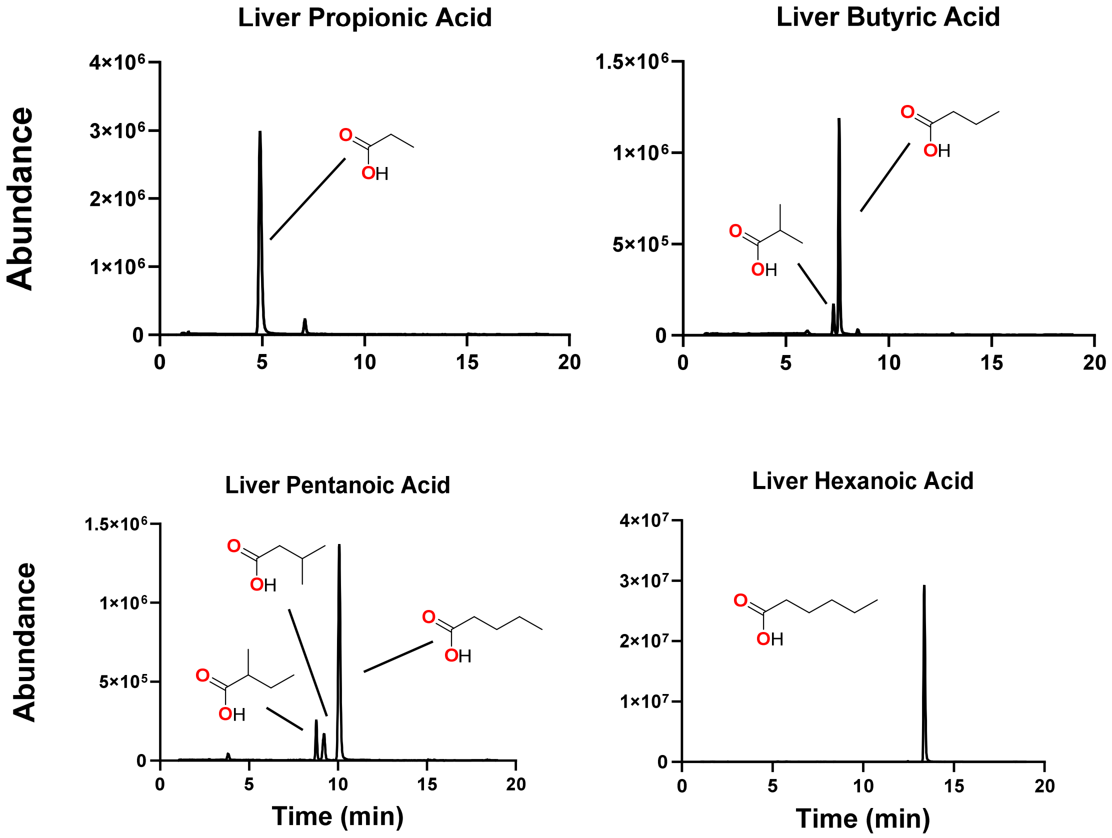

Supplementary Figure S2: Chromatograms (multiple ion monitoring) showing separation of C3, C4, C5 and C6 SCFA-4PyBA in pig liver. Peaks not labeled are likely isomers that we have yet to identify.

Figure S3.

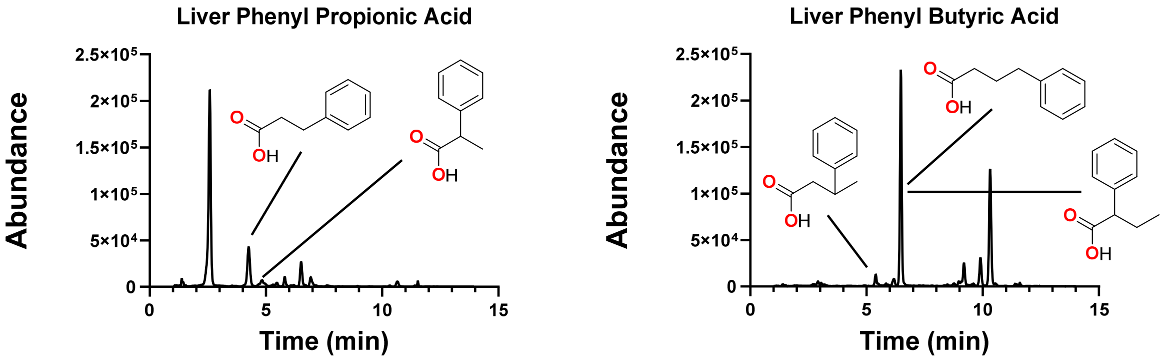

Supplementary Figure S3. Chromatograms (multiple ion monitoring) showing separation of C3 and C4 phenyl-SCFA-4PyBA in pig liver. No C5 or C6 phenyl-SCFA were detected. Peaks not labeled are likely isomers that we have yet to identify.

Figure S4.

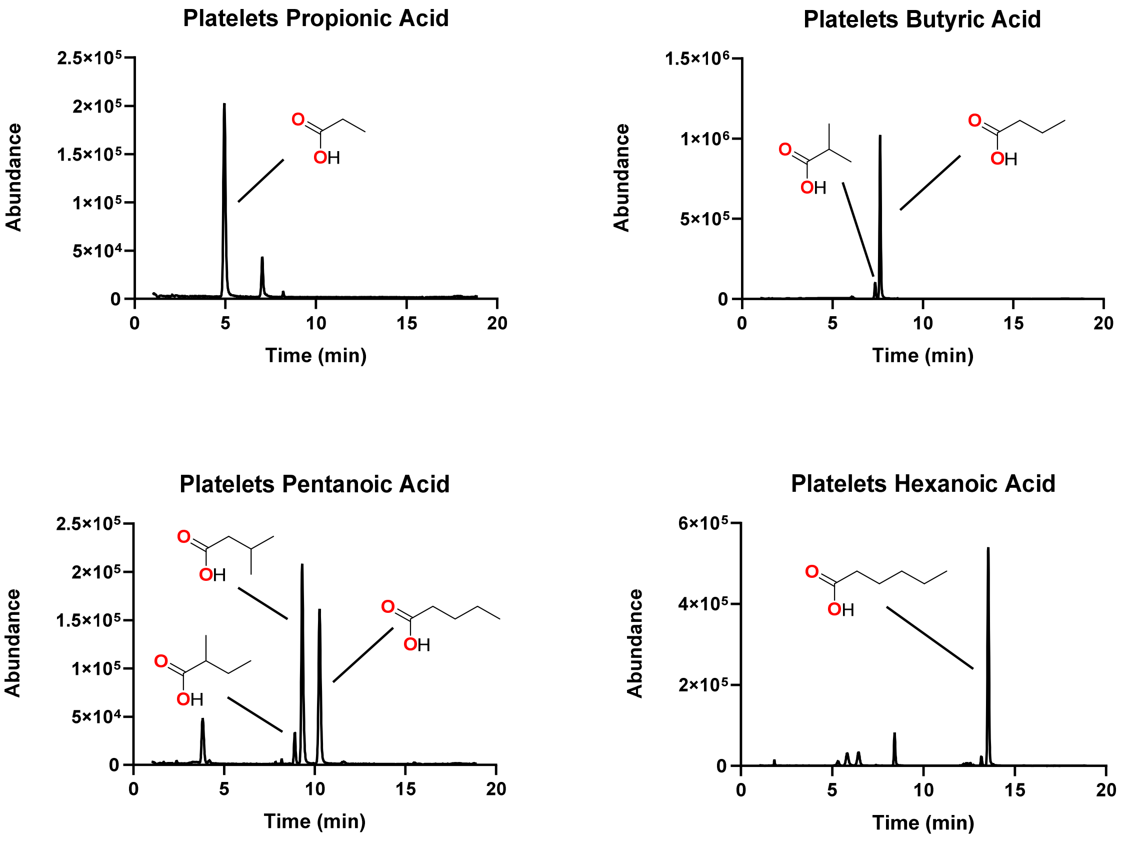

Supplementary Figure S4. Chromatograms (multiple ion monitoring) showing separation of C3, C4, C5 and C6 SCFA-4PyBA in rat platelets. Peaks not labeled are likely isomers that we have yet to identify.

Figure S5.

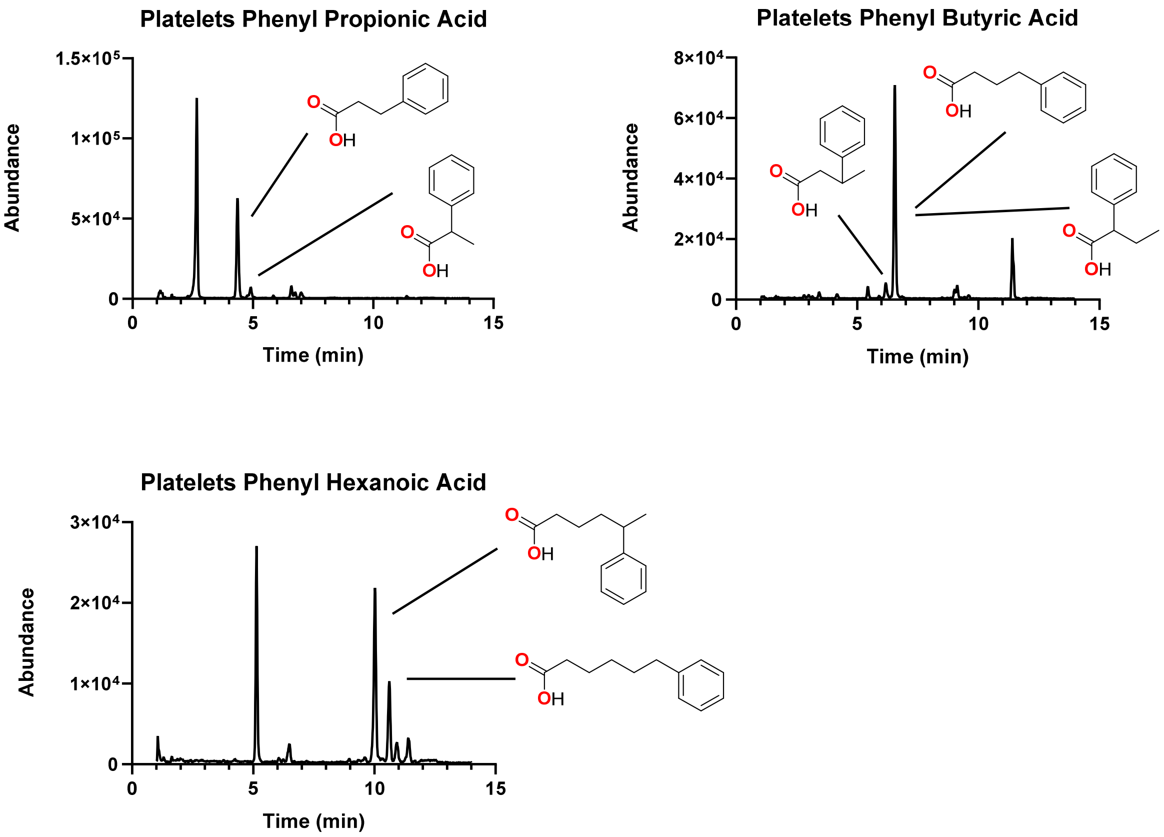

Supplementary Figure S5. Chromatograms (multiple ion monitoring) showing separation of C3, C4 and C6 phenyl-SCFA-4PyBA in rat platelets. No C5 phenyl-SCFA were detected. Peaks not labeled are likely isomers that we have yet to identify.

Figure S6.

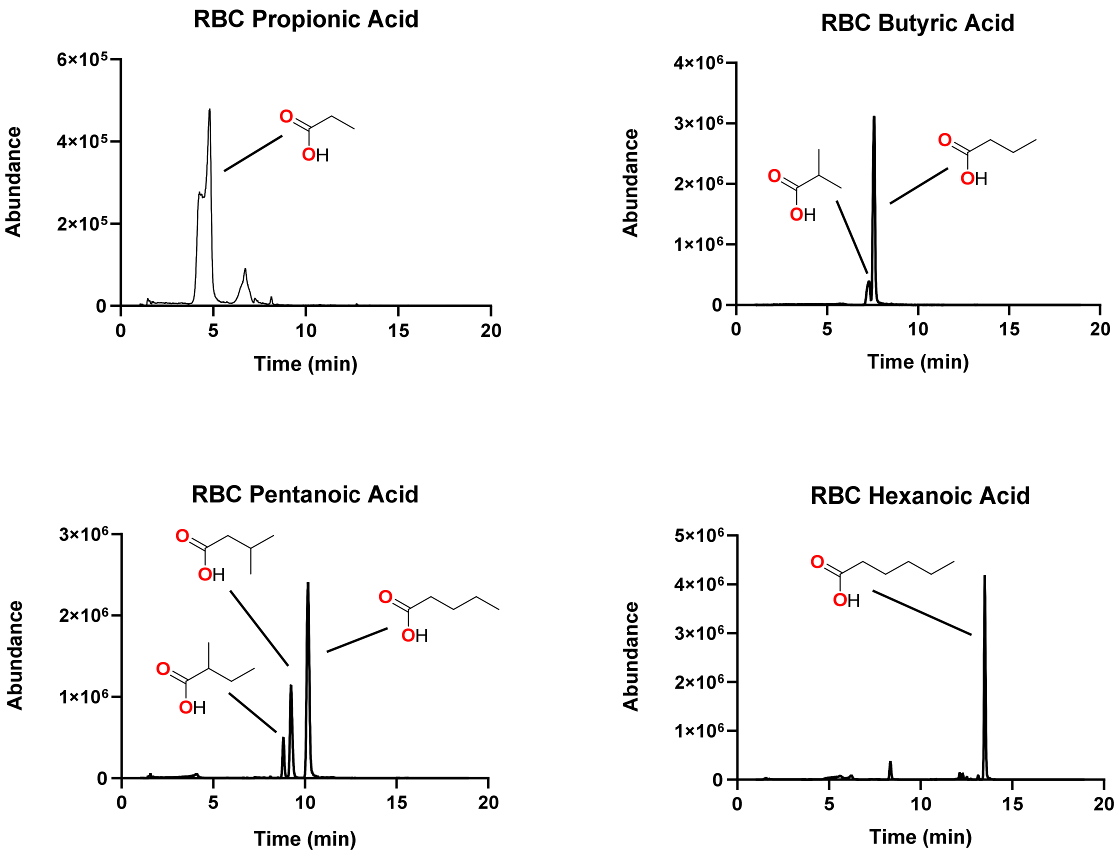

Supplementary Figure S6. Chromatograms (multiple ion monitoring) showing separation of C3, C4, C5 and C6 SCFA-4PyBA in rat red blood cells (RBC). Peaks not labeled are likely isomers that we have yet to identify.

Figure S7.

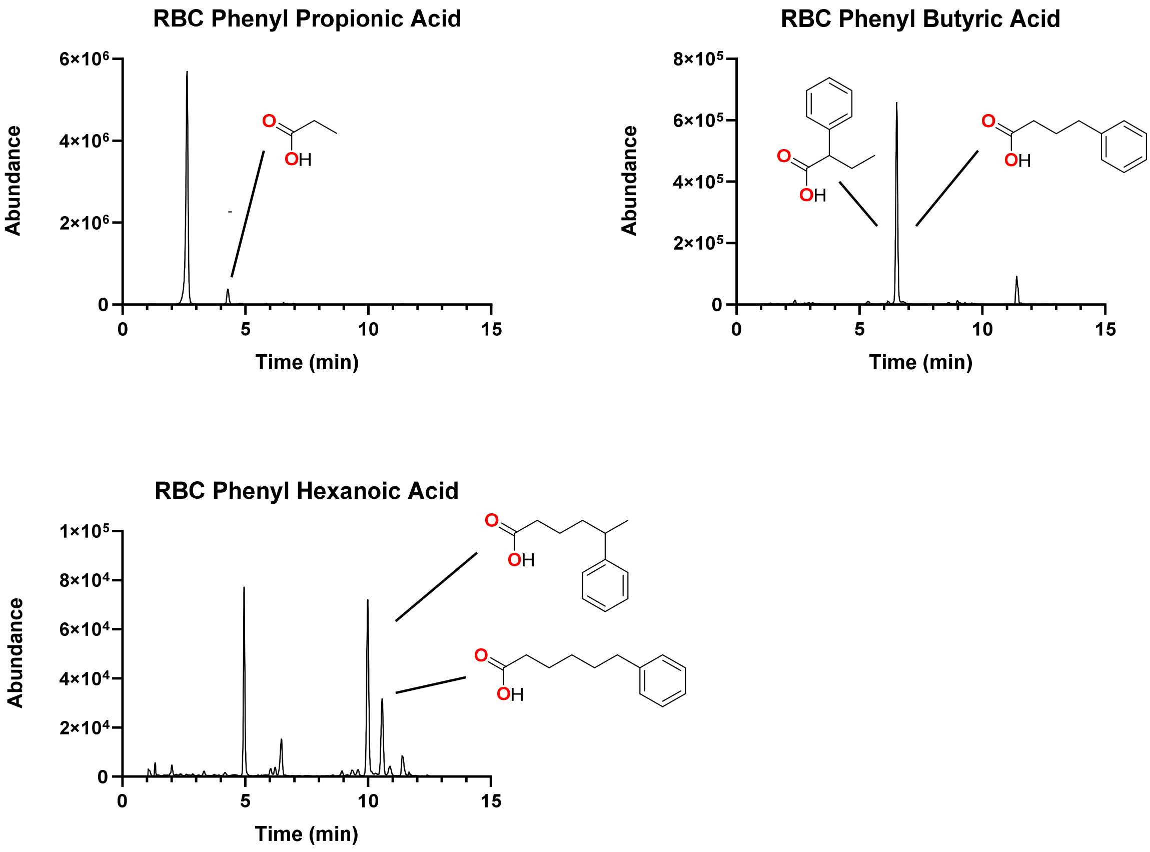

Supplementary Figure S7. Representative chromatograms (multiple ion monitoring) showing separation of C3, C4 and C6 phenyl-SCFA-4PyBA in rat RBC. No C5 phenyl-SCFA were detected. Peaks not labeled are likely isomers that we have yet to identify.
